# Delineation of attenuation of oxidative stress and mutagenic stress by *Murraya exotica* L. leaves

**DOI:** 10.1186/s40064-016-2709-0

**Published:** 2016-07-11

**Authors:** Davinder Kaur, Arvinder Kaur, Saroj Arora

**Affiliations:** Department of Botanical and Environmental Sciences, Guru Nanak Dev University, Amritsar, Punjab 143005 India

**Keywords:** Antioxidant activity, Antimutagenic activity, Epicatechin, *Murraya exotica*, Total phenolic content

## Abstract

**Background:**

*Murraya exotica* L., a member of family Rutaceae is rich in biologically active secondary metabolites and also known for its ethnobotanical importance. With this in mind, the plant was chosen and leaves were extracted sequentially to obtain ethanol, hexane, chloroform, ethyl acetate, *n*-butanol and aqueous fractions. The extract/fractions were evaluated for total phenolic and flavonoid content by spectrophotometric methods. The UHPLC technique was employed for profiling the different polyphenolic compounds present in the extract/fractions. Furthermore, the extract/fractions were analysed for the antioxidant and antimutagenic activities using different in vitro assays.

**Results:**

It was observed that, among the different extract/fractions, ethyl acetate fraction exhibited maximum total phenolic content i.e. 119.6 mg of GAE/g whereas chloroform fraction showed maximum total flavonoid content i.e. 323.5 mg of RE/g. Likewise, UHPLC method also showed maximum number as well as amount of polyphenolic compounds in ethyl acetate fraction. It was further analyzed that the same fraction exhibited the maximum radical scavenging activity in all antioxidant assays. In Ames assay, ethyl acetate fraction again showed maximum antimutagenic activity of 97.70 % against the 2-aminofluorene mutagen in TA100 tester strain of *Salmonella typhimurium*.

**Conclusion:**

Overall, among all the extract/fractions, ethyl acetate fraction was observed to be highly effective in scavenging the free radical as well as reducing the mutagenic effect of the mutagen. The maximum bioactivities of the ethyl acetate fraction may be linked to the presence of high number of polyphenolic compounds as shown by spectrophotometric as well as UHPLC methods.

## Background

Various degenerative diseases are known to be caused by the acquisition of cascade of mutations provoked by mutagens. However, many mutagens may act through the endogenous generation of reactive oxygen species which interact with the nucleotides, disulphide bonds and polyunsaturated fatty acids. These interactions support the execution of oxidation process of biological compounds and therefore, act as initiators of various degenerative diseases such as cardiovascular, emphysema, inflammatory diseases, cataracts etc. To combat the devastating effect of reactive oxygen species, cellular system has evolved many endogenous enzymatic antioxidants like superoxide dismutase, glutathione, catalase, glutathione peroxidases and reductase as well as non-enzymatic antioxidants like vitamin C, tocopherols and tocotrienols etc. (Devi et al. 2015). However, in many conditions, exogenous supply of antioxidants becomes essential due to the depletion of endogenous antioxidants by the overproduction of reactive oxygen species (Robin et al. 2015). In addition to above, the mutagenic activities of carcinogens are also due to the generation of free radicals. As a result, the exploration of antioxidant potential of compounds carries great therapeutic significance (Del-Toro-Sánchez et al. 2014). Many synthetic antioxidants have the limited availability, affordability and harmful side effects which results in shifting the interest towards the natural products that are capable of neutralizing the free radicals without any side effects (Wong-Paz et al. 2015). Natural based antioxidants are believed to be driven out the harmful effects of oxidative damage especially by acting as enzyme inhibitors, or synergists, peroxide decomposers, singlet and triplet quenchers (Zahin et al. 2013). The antioxidant rich medicinal plants as known to be the reservoir of bio-protective compounds, exerts their effects to scavenge the free radicals via inducing phase II enzymes (Devi et al. 2015). Therefore, efforts are needed to explore the medicinal plant for the presence of protective compounds (Zahin et al. 2013).

*Murraya exotica* L. (commonly known as Chinese box or Jasmine orange) is an evergreen shrub, usually 2–3 m in height. *M. exotica* is the native of continental tropical Asia. It is extensively grown as an ornamental plant and for fencing the gardens (Ghani, 1998). The leaves of *M. exotica* have been reported to contain several secondary metabolites such as alkaloids, coumarins, flavonoids, phytosterols and dipeptides (Desoky et al. 1992; Barik and Kunda 1987; Barik et al. 1983; Ito and Furukawa 1987; Bishay et al. 1987; Desoky 1995; Ahmad and Begum 1987). The leaves have been reported to possess various properties such as stimulant, abortive and also used to treat dysentery, cuts, body aches, joint pain, diarrhea, venereal diseases (Kinoshuta and Fireman 1996; Parrotta 2001; Xiao and Wang 1991). Kong et al. (1985) reported the contraceptive properties of this plant. Yuehchukene, an alkaloid isolated from this plant showed the significant anti-implantation effects in female mice, when given orally or subcutaneously. Low dose of yuehchukene were also reported to have anti-tumor effects (Leung et al. 2000). A coumarin i.e. murrangatin, derived from the leaves showed antithyroid property (Khare 2007).

Keeping all in mind coupled with the fact that *M. exotica* is a rich source of polyphenolic compounds and has also not been much explored for the antioxidant and antimutagenic activity, the present study was conducted.

## Methods

### Extraction and phytochemical analysis

Fresh leaves (disease free) of *M. exotica* were collected in the month of December, 2011 from Mata Kaulan botanical garden of Guru Nanak Dev University campus, Amritsar. Plant specimen (accession no. 7315) was identified and deposited in herbarium of Department of Botanical and Environmental Sciences, Guru Nanak Dev University, Amritsar. Air dried leaves powder was extracted via using sequential extraction method to obtain the hexane, chloroform, ethyl acetate, *n*-butanol and aqueous fractions. The total phenolic and flavonoid content of extract/fractions was determined spectrophotometrically following the method of Singleton and Rossi (1965) and Kim et al. (2003) respectively. These extract/fractions were further subjected to UHPLC analysis for the profiling and quantification of polyphenolic compounds which was performed on Nexera UHPLC (Shimadzu) system equipped with C-18 Column. For this, 10 µl of sample [10 mg extract or fraction/ml of methanol (HPLC Grade)] was filtered through 0.22 micron filters and injected into column with column temperature: 27 °C, flow rate: 1 ml/min, mobile phase: water (A) and methanol (B), run time: 26 min. Identification of compounds was achieved through the comparison of their retention time and UV spectra with standards whereas quantification was done by using linear gradient elution method.

### Antioxidant activity

The antioxidant activity of extract/fractions of *M. exotica* was measured in terms of hydrogen donating or radical scavenging ability via using DPPH radical scavenging assay (Blois 1958) and ABTS radical cation decolorization assay given by Re et al. (1999).

The reductive capacity of iron (III) was determined using the method of Oyaizu (1986), Cu(II)-Nc by CUPRAC assay given by Apak et al. (2007) and the molybdate ion by the method described by Prieto et al. (1999).

Hydroxyl radical scavenging ability of extract/fractions was determined by using DNA nicking assay which protect the supercoiled pBR 322 plasmid DNA from devastating effects of hydroxyl radicals generated by Fenton’s reagent as described by Lee et al. (2002). Briefly, 0.3 μl of plasmid DNA was mixed with 10 μl of Fenton’s reagent (30 mM H_2_O_2_, 50 μM ascorbic acid, and 80 μM FeCl_3_) followed by the addition of 10 μl of extract/fraction. The final volume of the mixture was adjusted to 20.5 μl with distilled water. After incubate the reaction mixture for 30 min at 37 °C, the DNA was loaded on a 1 % agarose gel (prepared by dissolving 0.5 g of agarose in 50 ml of 1X TBE Buffer followed by ethidium bromide staining) and electrophoresis was accomplished. Rutin was taken as a positive control and the densitometric analysis was done by using AlphaEaseFC 4.0 software for determining the hydroxyl radical scavenging ability of extract/fraction.

### Antimutagenicity assay

The Ames Salmonella histidine reversion assay described by Maron and Ames (1983) with slight modifications given by Bala and Grover (1989) was used to elucidate the antimutagenic activity of different extract/fractions for TA98 (frame shift mutation) and TA100 (base pair substitution) tester strains of *Salmonella typhimurium*. TA98 and TA100 strains of *S. typhimurium* were procured from Prof. B.N. Ames, University of California, Berkeley. Different concentrations of extract/fractions (100, 250, 500, 1000 and 2500 µg/0.1 ml of DMSO/plate) were tested against direct acting mutagens i.e. nitro-o-phenylenediamine (NPD, 20 µg/0.1 ml DMSO/plate) and sodium azide (NaN_3_, 2.5 µg/0.1 ml DMSO/plate) for TA98 and TA100, respectively by using two experimental procedures, co-incubation and pre-incubation experiment. In the co-incubation experiment, a mixture of 0.1 ml of bacterial culture, 0.1 ml of mutagen, 0.1 ml of different concentrations of extract/fractions was added to 2 ml of top agar (0.5 % NaCl, 0.5 % Agar and 0.5 mM Histidine/Biotine solution) which was then poured onto minimal agar plates. In the pre-incubation experiment, the mixture of 0.1 ml of mutagen, 0.1 ml of different concentrations of extract/fraction and 0.5 ml of S9 mix (in case of indirect mutagen) was incubated for 30 min at 37 °C. After incubation, the mixture along with 0.1 ml of culture was added to 2 ml top agar and then poured onto minimal agar plates. Concurrently, spontaneous (only 0.1 ml bacterial culture in 2 ml top agar), positive control (0.1 ml bacterial culture along with 0.1 ml mutagen in 2 ml top agar) and negative control (0.1 ml bacterial culture along with 0.1 ml extract/fraction concentration in 2 ml top agar) were also kept. Simultaneously, the antimutagenic activity of extract/fractions was also assessed against indirect acting mutagen 2-Aminofluorene (2AF, 20 µg, 0.1 ml DMSO/plate) in the presence of cytochrome based P450 metabolic system (S9 mix) for TA98 and TA100. The minimal agar plates were incubated at 37 °C for 48 h and number of histidine-independent revertant colonies was scored. The antimutagenicity was expressed as percentage decrease of reverse mutations as follow:

$${\text{Inhibition}}\,\left( \% \right) = \left( {\left( {{\text{a}} - {\text{b}}} \right)/\left( {{\text{a}} - {\text{c}}} \right)} \right)*100$$

where a = number of histidine revertants induced by mutagen (positive control), b = number of histidine revertants induced by mutagen in the presence of extract/fraction, c = number of histidine revertants induced in the presence of extract/fraction alone and solvent (negative control). All the experiments were performed in triplicates.

### Statistical analysis

The results are shown as means of the triplicates with standard error. Furthermore, statistical significance of the data was analyzed using the analysis of variance (one-way ANOVA) and Tukey’s multiple comparison tests to determine the interactions and differences at 5 % level of significance.

## Results

### Extraction and phytochemical analysis

**Fig. 1:**
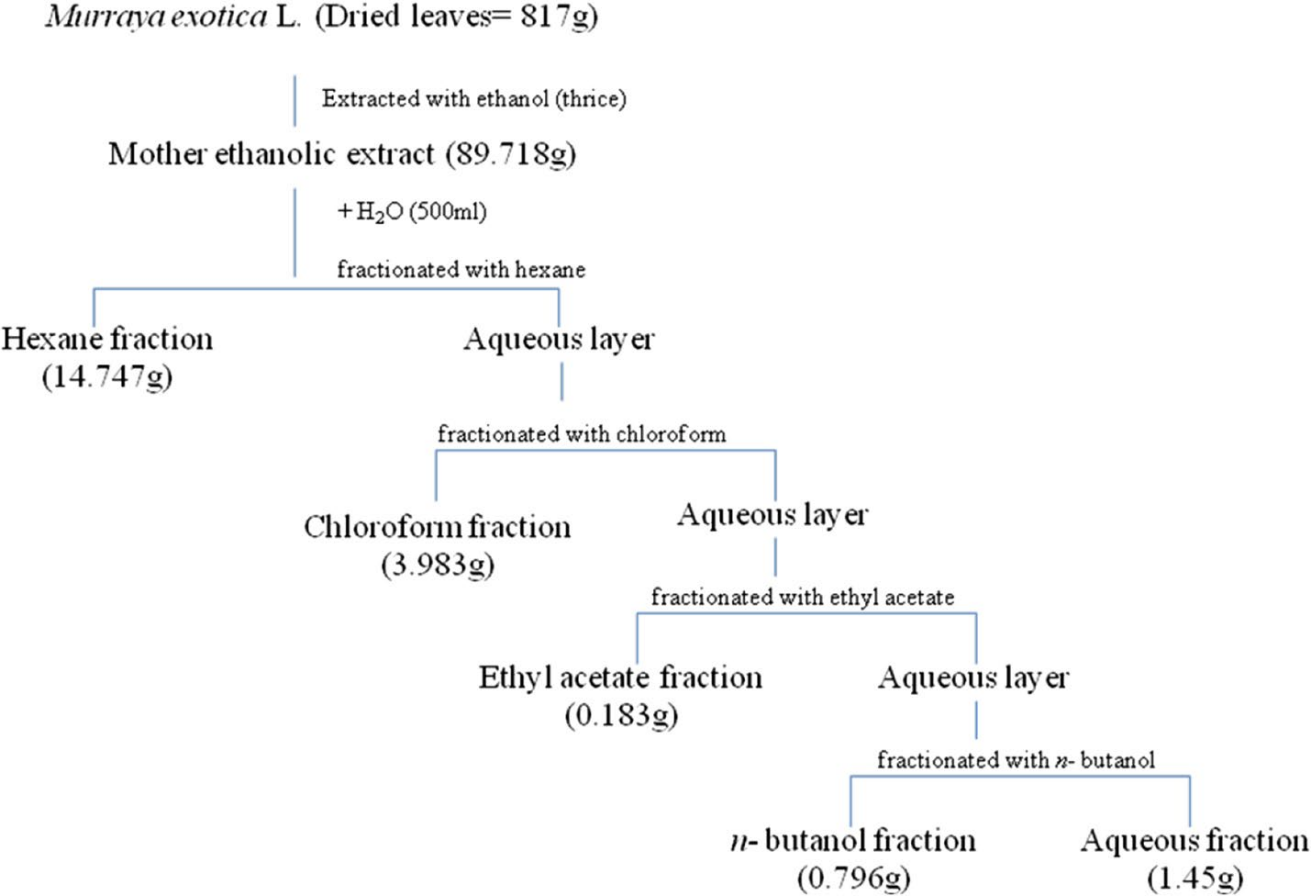
Extraction and fractionation sketch of *M. exotica* leaves

In the present study, leaves of *M. exotica* were extracted with ethanol to obtain the mother ethanolic extract (89.718 g). The ethanolic extract (22 g) yielded 67.03, 18.10, 0.83, 3.62 and 6.59 % of fraction when fractionated with hexane, chloroform, ethyl acetate, *n*-butanol and aqueous respectively as shown in Fig. 1 with maximum % yield of hexane fraction. The total phenolic content was found highest in ethyl acetate fraction i.e. 119.6 mg GAE/g and followed by *n*-butanol > chloroform > hexane > ethanol > aqueous fraction. Total phenolic content was determined by using the standard curve equation of y = 0.0005x + 0.0377, R^2^ = 0.99. Polyphenolic compounds like flavonoids have been labelled as “high level natural antioxidants” based upon their ability to scavenge free radicals and active oxygen species. Total flavonoid content was calculated from standard curve equation of y = 0.0006x + 0.0454 with R^2^ = 0.99. The highest flavonoid content of 323.5 mg RE/g was measured in chloroform fraction as compared to other extract/fractions. The total flavonoid content in other extract/fractions was found in the order of ethanol > hexane > ethyl acetate > *n*-butanol > aqueous fraction (Table 1).

**Table 1:** Extraction yield, total phenolic content (TPC) and total flavonoid content (TFC) in extract/fractions of *M. exotica* leaves

Extract/fractions	Extraction yield (%)	TPC (mg of GAE/g)	TFC (mg of RE/g)
Ethanol	10.98	29.6	282.66
Hexane	67.03	31.6	239.33
Chloroform	18.10	83.6	323.5
Ethyl acetate	0.83	119.6	96
*n*-butanol	3.62	99.6	54.33
Aqueous	6.59	27.6	19.33

The UHPLC analysis for polyphenolic compounds showed that sequential extraction of leaves results in the extraction of the different amount as well as number of polyphenolic compounds in different extract/fractions. The maximum number of polyphenolic compounds with high content of epicatechin (133.63 ppm) was identified in ethyl acetate fraction of leaf extract. A chromatograph obtained for other extract/fractions showed the presence of highest concentration of *tert*-butyl hydroquinone (35.50 and 175.44 ppm) in ethanol and *n*-butanol fractions respectively, kaempferol (186.44 ppm) in hexane fraction, rutin (94.33 ppm) in chloroform fraction and chlorogenic acid (18.85 ppm) in aqueous fraction along with other polyphenols in minute concentrations as depicted in Table 2.

**Table 2:** UHPLC analysis of different extract/fractions of *M. exotica* (concentration in ppm)

S. no.	Standard compounds		Ethanol extract	Hexane fraction	Chloroform fraction	Ethyl acetate fraction	*n*-butanol fraction	Aqueous fraction
1.	Gallic acid	664.017				0.179	1.059	0.267
2.	Catechin	665.645				4.605	12.343	17.229
3.	Chlorogenic acid	333.184	0.308			5.544	29.72	*18.859*
4.	Epicatechin	665.418	3.483	0.389	58.787	*133.63*	13.41	0.275
5.	Caffeic acid	332.865	0.154		0.175	2.911	13.376	2.985
6.	Umbelliferone	333.016	0.371	0.371	2.102	73.238	8.569	0.22
7.	Coumaric acid	332.710			0.096	4.202	1.194	0.416
8.	Rutin	666.242	11.102	4.984	*94.331*	8.586	2.867	
9.	Ellagic acid	333.193		0.323	8.078	11.348	17.703	
10.	Tert-Butyl hydroquinone	332.743	*35.507*	13.332	68.882	62.351	*175.442*	1.178
11.	Quercetin	332.449	0.817	0.194	0.972	11.777		
12.	Kaempferol	332.296	3.966	*186.443*	6.176			

Figures in italic showed the presence of highest amount of particular phenolic compound in respective extract/fraction

### Antioxidant activity

The antioxidant potential of different extract/fractions was examined by employing the different in vitro antioxidant assays viz DPPH, ABTS, FRAP, CUPRAC and molybdate ion reduction assays.

**Fig. 2:**
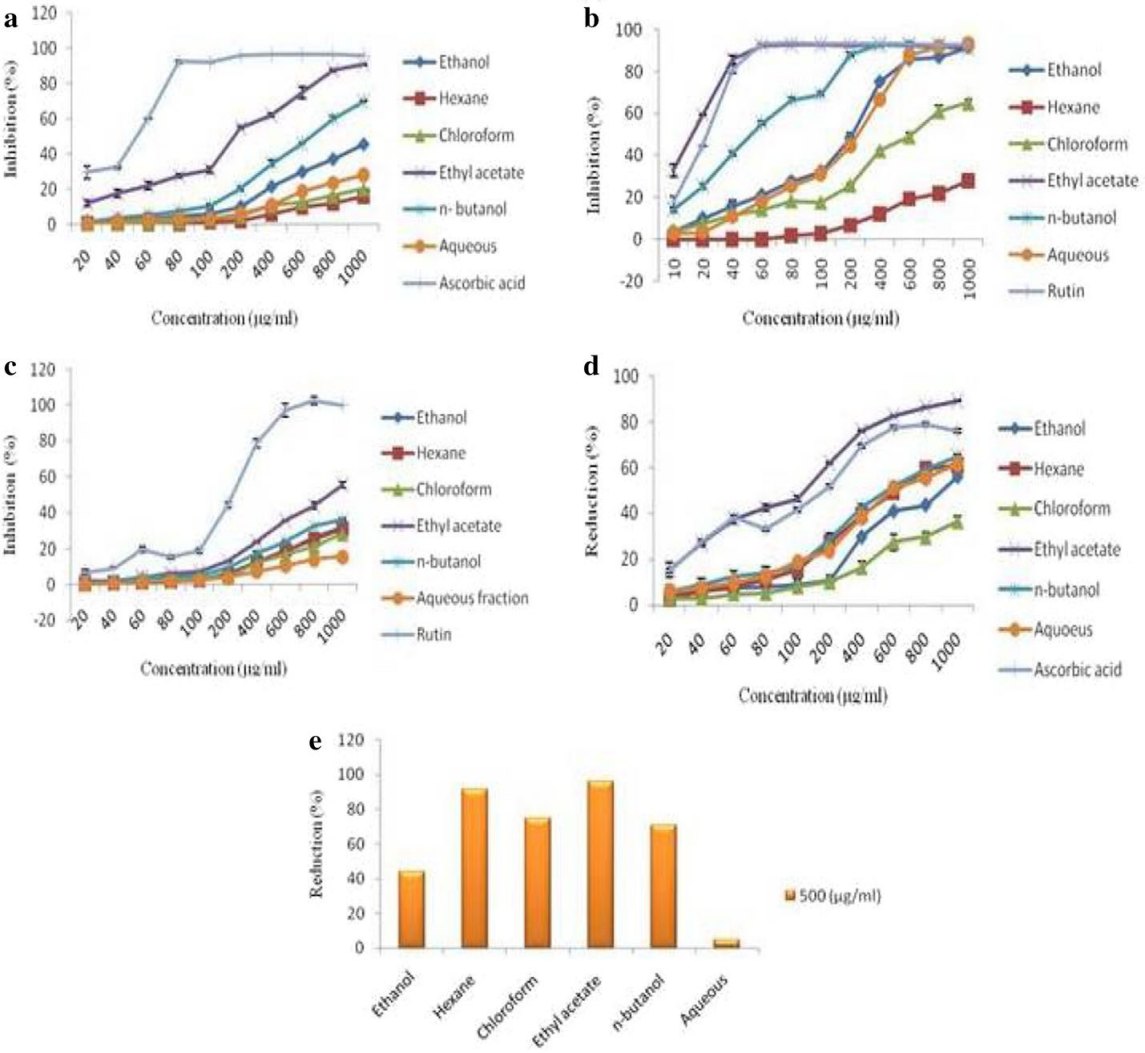
Antioxidant activity of different concentrations of extract/fractions of *M. exotica* leaves accessed by DPPH radical scavenging assay (**a**), ABTS radical cation decolorization assay (**b**), FRAP assay (**c**), Cupric-Ion-Reducing Antioxidant Capacity (CUPRAC) assay (**d**), Molybdate ion Reduction assay (**e**). Data expressed as mean of % inhibition ± SE of triplicates

The decolorizing activity following the trapping of unpaired electron of DPPH and ABTS radical cation was used as a measure of free radical scavenging activity (Fig. 2a, b). The radical scavenging activity of extract/fractions exhibited the dose dependent response in DPPH assay. The most effective free radical scavengers were found in ethyl acetate fraction with 90.77 % decolorization at concentration of 1000 µg/ml whereas ascorbic acid (positive control) showed 91.96 % of decolorization at 100 µg/ml concentration. Likewise, *n*-butanol, ethanol, aqueous, chloroform and hexane fractions exhibited the 70.05, 45.28, 28.27, 20.25 and 16.09 % of decolorization activity respectively. In ABTS radical cation assay, all extract/fractions showed dose dependent free radical scavenging activity with highest potential (92.80 %) of ethyl acetate fraction which was found to be comparable to positive control (rutin). The ABTS radical cation decolorization activity was found in the order of ethyl acetate > *n*-butanol > ethanol > aqueous > chloroform, and least in hexane fraction.

The reducing potential as exhibited by extract/fractions is shown in Fig. 2c–e. The Fe(III) reducing activity of all the extract/fractions was found to be dose dependent (Fig. 2c). Furthermore, at 1000 µg/ml concentration, the ethyl acetate fraction demonstrated maximum reducing potential of 55.48 %. Whereas, other extract/fractions also showed the reducing ability in the order of *n*-butanol (36.07 %) > ethanol (31.88 %) > hexane (31.08 %) > chloroform (28.05 %) and least in the aqueous fraction (15.69 %) at same concentration. Similarly, in cuprac assay, ethyl acetate fraction again exhibited the highest reducing power potential (89.28 %) as compared to other extract/fractions at their highest concentration (Fig. 2d). However, rutin which was used as a positive control had comparable activity to ethyl acetate fraction and superior activity than other extract/fractions. In case of Molybdate ion reducing ability, the antioxidant activity was measured by using the standard curve of Gallic acid with regression equation of y = 0.002x + 0.086 (R^2^ = 0.94). The antioxidant activity by Molybdate ion reducing ability assay indicated that the highest antioxidant potential was determined in ethyl acetate fraction i.e. 95.83 mg GAE/g dry weight of extract/fractions which was followed by hexane (91.5) > chloroform (75) > *n*-butanol (70.83) > ethanol (44.33) and aqueous fraction (4.66 mg GAE/g) at 500 µg/ml concentration as shown in Fig. 2e.

**Fig. 3:**
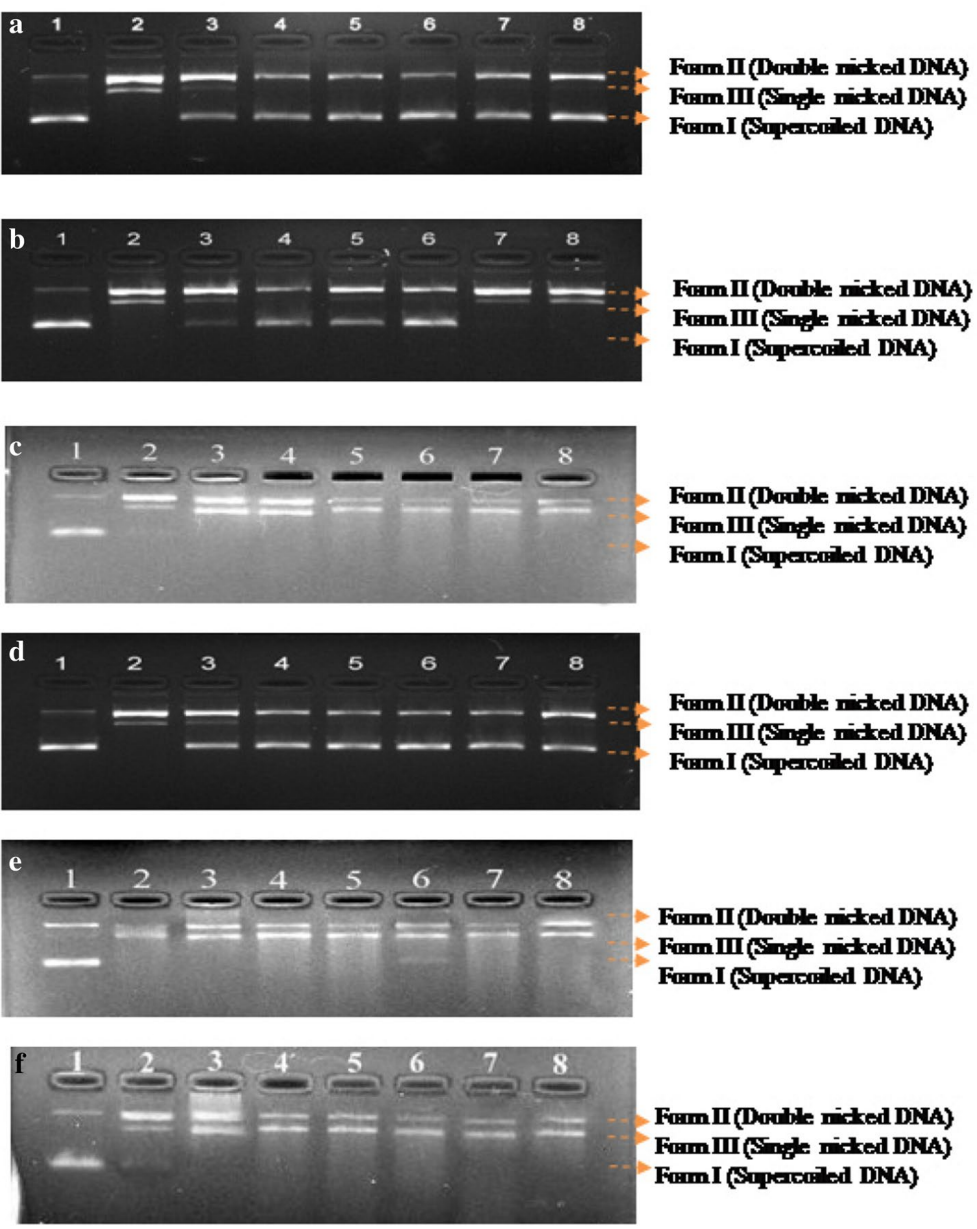
DNA nicking assay for non site-specific ˙OH scavenging activity of ethanol extract (**a**), hexane (**b**), chloroform (**c**), ethyl acetate (**d**), *n*-butanol (**e**), aqueous fractions (**f**) of *M. exotica* leaves. *Lane 1* represents: pBR322 DNA + distilled water; *lane 2* pBR322 DNA + distilled water + Fenton Reagent (FR); *lane 3* pBR322 DNA + FR + Rutin; *lane 4–8* pBR322 DNA + FR + Extract/fraction (1000, 800, 600, 400 and 200 µg/ml respectively)

The hydroxyl radical scavenging ability of different concentrations of extract/fractions was observed by DNA nicking assay. As shown in Table 3 and Fig. 3, the presence of extract/fractions retained the maximum amount of supercoiled DNA in its native form (form I) by scavenging the hydroxyl radicals as generated by Fenton’s reagent. From the densitometric analysis, it was found that ethyl acetate fraction significantly conserved the 52.8 % of DNA in its native form by reducing the hydroxyl mediated breaking, conversion of supercoiled DNA into open circular (form II) and linear (form III) via scavenging the hydroxyl radicals in a dose dependent manner and thus providing the significant protection against oxidative stress.

**Table 3:** Densitometric analysis of DNA in plasmid nicking assay of extract/fractions of *M. exotica* leaves

Form of DNA	Amount of DNA (%)^a^							
	1	2	3	4	5	6	7	8
Ethanol extract								
Form I	69.8	–	30.7	52.1	51.8	49.8	43.8	41.5
Form II	30.2	71.4	54.9	40.2	40.5	42.5	50.0	53.0
Form III	–	28.6	14.4	7.8	7.7	7.7	6.2	5.5
Hexane fraction								
Form I	81.6	–	17.7	43.2	41.2	42.0	–	–
Form II	18.4	72.6	66.5	46.4	53.3	43.0	79.6	73.4
Form III	–	27.4	15.9	10.4	8.9	15.0	20.1	26.6
Chloroform fraction								
Form I	68.3	–	–	–	–	–	–	–
Form II	31.7	63.15	48.1	49.1	55	55.4	54.2	55.7
Form III	–	36.85	51.9	50.9	45	44.6	45.8	44.3
Ethyl acetate fraction								
Form I	79.1	–	39.1	58.2	55.9	55.2	52.9	40.3
Form II	20.9	76.3	45.8	35.4	35.2	36.0	38.1	49.7
Form III	–	23.7	15.1	6.4	8.9	8.8	9.0	10.0
*n*-butanol fraction								
Form I	69	–	–	–	–	–	–	–
Form II	31	51.9	45.2	47.3	48.6	51	52.6	55.3
Form III	–	48.1	54.8	52.7	51.4	49	47.4	44.7
Aqueous fraction								
Form I	72.4	–	–	–	–	–	–	–
Form II	27.6	65.1	50.4	51.1	51.6	52.8	53.8	56.3
Form III	–	34.9	49.6	48.9	48.4	47.2	46.2	43.7

^a^Lane 1: pBR322 DNA + distilled water; lane 2: pBR322 DNA + distilled water + Fenton Reagent (FR); lane 3: pBR322 DNA + FR + Rutin; lane 4–8: pBR322 DNA + FR + Extract/fraction (1000, 800, 600, 400 and 200 µg/ml respectively)

### Antimutagenic activity

On the basis of promising antioxidant and reducing activity, the different concentrations of extract/fractions were tested to explore the antimutagenic activity of *M. exotica*. All extract/fractions at different concentrations (100, 250, 500, 1000 and 2500 µg/0.1 ml/plate) were found to be non-toxic and non-mutagenic for TA98 and TA100 strains in the absence as well as presence of S9 mix.

**Fig. 4:**
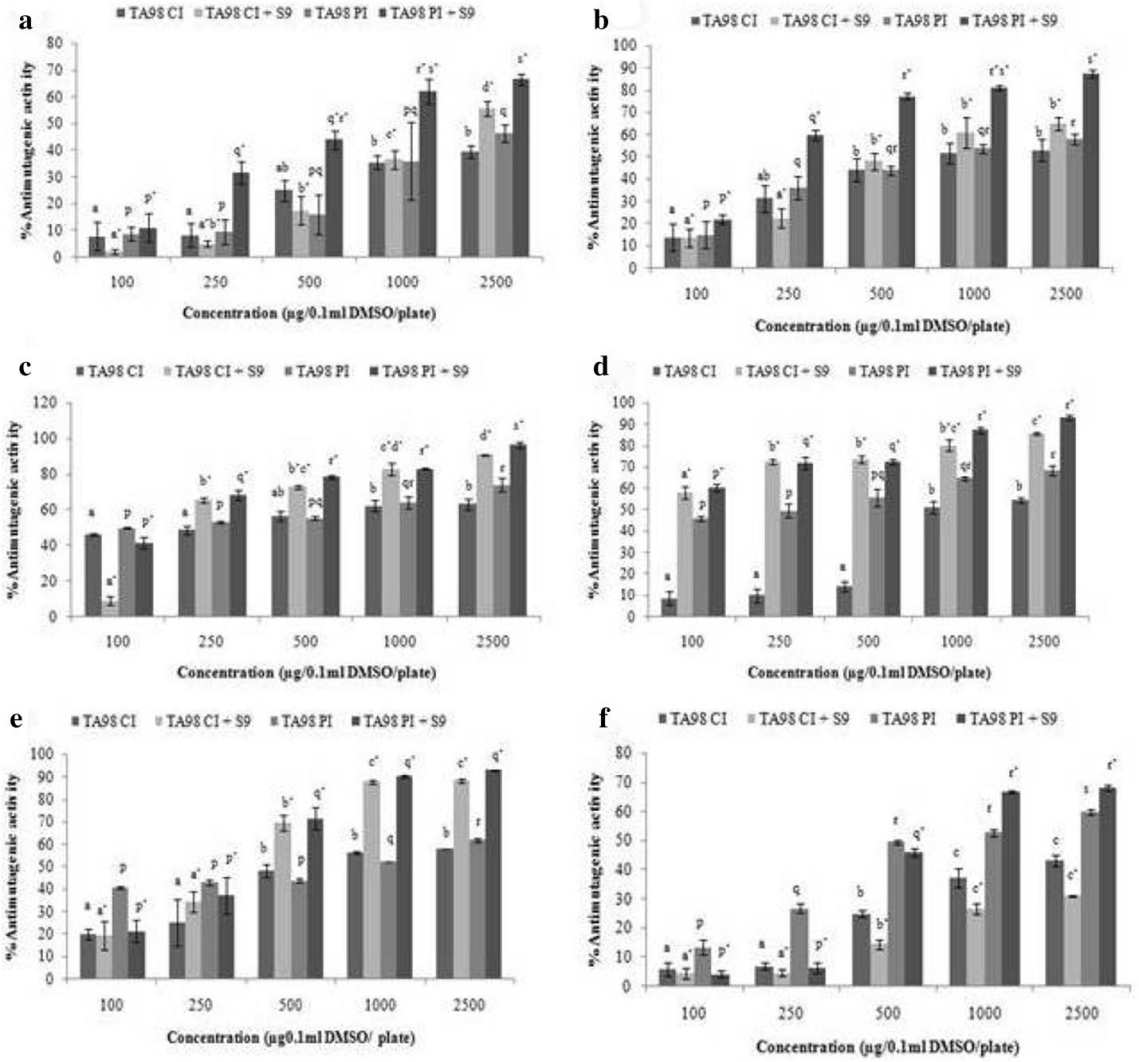
Graphical representation of antimutagenic activity of ethanol extract (**a**), hexane (**b**), chloroform (**c**), ethyl acetate (**d**), *n*-butanol (**e**), aqueous fractions (**f**) for TA98 strain of *S. typhimurium* showing the comparison between co- and pre-incubation in the absence as well as presence of S9 mix in AMES assay. Data represented is the mean of % antimutagenic activity ± SE of the triplicates. Means followed by same letters are not significantly different and followed by different letters are significantly different at p ≤ 0.05 using HSD Multiple comparison test. *Letter* followed by “*asterisk*” shows the mean of % inhibition ± SE of the triplicates in the presence of S9 mix

**Fig. 5:**
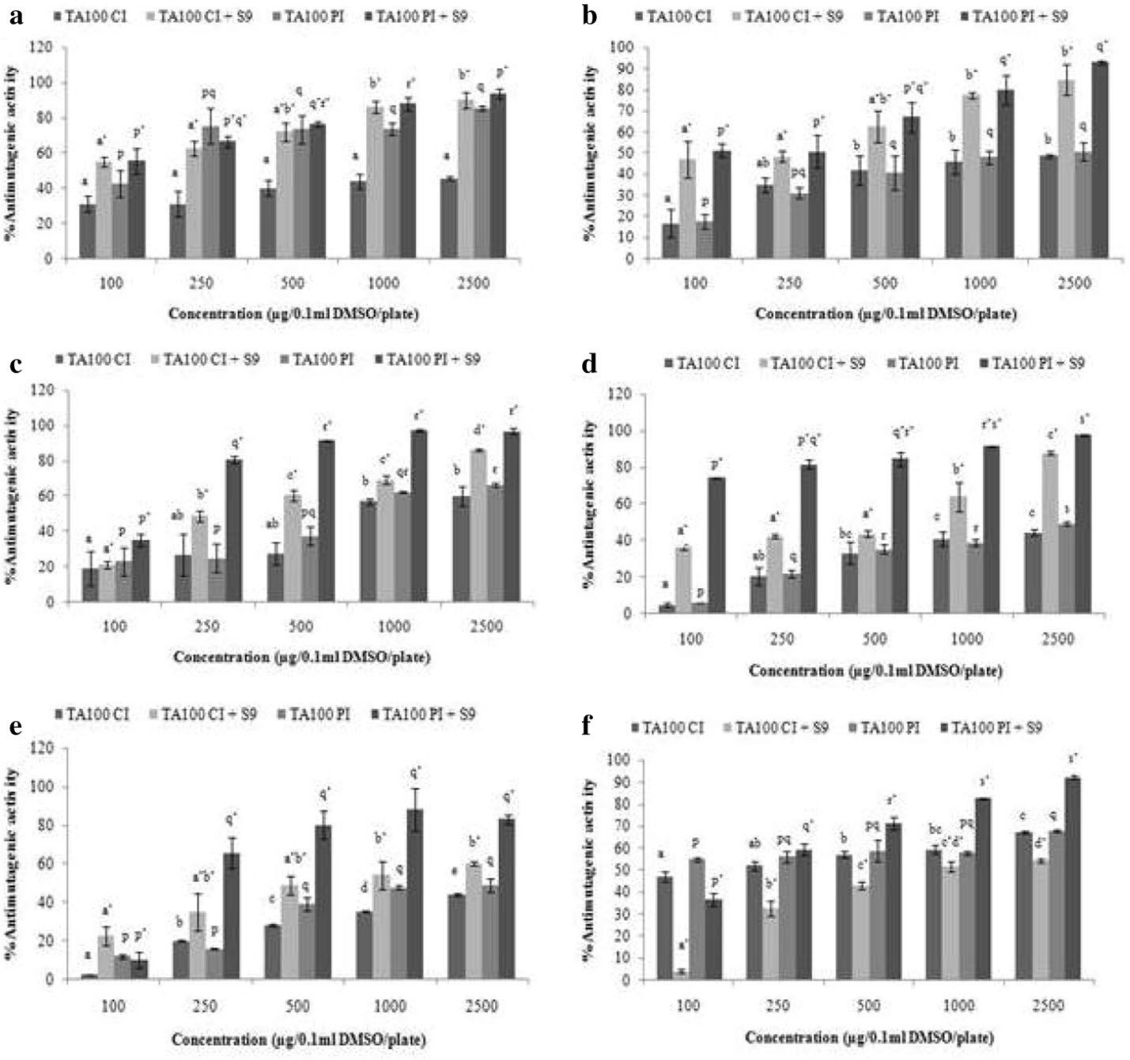
Graphical representation of antimutagenic activity of ethanol extract (**a**), hexane (**b**), chloroform (**c**), ethyl acetate (**d**), *n*-butanol (**e**), aqueous fractions (**f**) for TA100 strain of *S. typhimurium* showing the comparison between co- and pre-incubation in the absence as well as presence of S9 mix in AMES assay. Data represented is the mean of % antimutagenic activity ± SE of the triplicates. Means followed by same letters are not significantly different and followed by different letters are significantly different at p ≤ 0.05 using HSD Multiple comparison test. *Letter* followed by “*asterisk*” shows the mean of % inhibition ± SE of the triplicates in the presence of S9 mix

Against TA98, the antimutagenic response of chloroform fraction was found to be significant with 74.04 % decrease in NaN_3_-induced mutagenicity and 96.31 % decrease in 2-AF-induced mutagenicity (Fig. 4c). Linear regression was found to be strong with S9 (R^2^ = 0.938) as compared to without S9 (R^2^ = 0.920). On the contrary, the ethyl acetate fraction significantly (p ≤ 0.005) decrease the number of His^+^ revertants induced by sodium azide by 49.07 % (R^2^ = 0.934) and 2-AF by 97.70 % (R^2^ = 0.991, highest among all extract/fractions) for TA100 as depicted in Fig. 5d. The antimutagenic effect was found to be more pronounced in pre-incubation than co-incubation in the presence of S9 mix (Figs. 4, 5). The linear regression analysis showed the presence of strong correlation between the extract/fraction dose and antimutagenic response against the mutagen with the R^2^ value ranges from 0.701 to 0.991. The whole experimentation was also scrutinized statically for interaction and difference by two-way ANOVA which showed the significant difference between the different concentrations as well as different modes of experimentations (Pre and co-incubation mode) at 5 % level of significance.

## Discussion

Oxidative stress induced diseases can be prevented by the removal of free radicals from circulation via good chelators. Devi et al. (2015) revealed that the plant metabolites like polyphenols are effective free radical scavenges and can be easily metabolized inside the body without any harmful effects. So an attempt has been made to find out the phytoconstituents especially polyphenolic and flavonoids and then elucidate the antioxidant and antimutagenic activity of extract/fractions of *M. exotica*.

A critical analysis of results showed that ethyl acetate fraction was rich in polyphenolic compounds and exhibited the maximum antioxidant and antimutagenic activities. There is plethora of reports available in literature which corroborates the results obtained in present study. The studies conducted by Gautum et al. (2012) and Khatun et al. (2014) showed the presence of phenols and flavonoids in different species of Murraya. The total phenolic content and total flavonoid content was found to be varying in different extract/fractions due to fractionation of leaves with different solvents. In the present study, the ethyl acetate fraction of *M. exotica* leaves were found to have high total phenolic content which might be responsible for the maximum antioxidant activity in all tested assays. The study conducted by Zahin et al. (2013) also emphasizes the antioxidant potential of different extract/fractions of *M. koenigii* over the range of 0–120 µg/ml and found that benzene fraction exhibited the maximum antioxidant potential. A redox potential of the phenolic compounds that contributes to the antioxidant property mainly include free radical scavenging activity, transition-metal-chelating activity and singlet-oxygen-quenching activity (Ciz et al. 2010) and thus, involve in the termination of free radical chain reactions (Ningappa et al. 2008). At certain concentration of reactive oxygen species, the OH bond of polyphenols is broken and released the hydrogen ion which contributes towards the antioxidant potential by further quenching the nucleophilic radicals (Devi et al. 2015). So, the most significant radical scavenger determinant in the polyphenols mainly includes a free 3-hydroxyl group, 3′,4′-catechol structure and a 4-oxo group on the C ring. These structural properties are also present in epicatechin which might act as principle components in ethyl acetate fraction (high phenolic content) for its maximum activity in all antioxidant assays. Literature reviews also revealed a positive correlation between the phenolic content and the antioxidant activity in mushroom (Chang et al. 2007). A correlation was also found in agreement with the finding of Zhang et al. (2011) and Anagnostopoulou et al. (2005) that showed the potent antioxidant ability of seventy polymethoxylated flavonoids (PMFs) isolated from leaves of *M. exotica.*

Oxidative damage in pBR322 DNA strands induced by hydroxyl radicals generated by Fenton reagent results in the formation of nicked circular form (form II, formed by single stranded scission) and linear form (form III, formed by double stranded breaks). By chelating the redox-active transition metal ions, polyphenols might be acts as protecting agents against the devastating effects of oxidative damage (Prakash et al. 2007). Polyphenols with catechol, 4-oxo and OH group also inhibited the Fenton induced oxidation via scavenging the radicals (Ciz et al. 2010). The study conducted by Kumar et al. (2010) also revealed that the methanol extract of *M. exotica* with high phenolic (510 mg GAE/g dry weight of extract) and flavonoid content (55.4 mg RE/g dry weight of extract) was found to be effective in plasmid nicking assay. Similarly, in our study, the ethyl acetate fraction with high phenolic content showed the significant protection against damage induced by hydroxyl radicals.

The difference in antioxidant activity of different extract/fractions might be ascribed to the presence of different chemical constituents. Overall, antioxidant activity of extract/fraction may be the result of synergic interactions of polyphenolic compounds with one another, and/or other compounds present in the extract/fraction (Hatano et al. 1989; Laranjinha et al. 1995; Van-Acker et al. 1998).

Mutations are considered as the early step in carcinogenesis. Endogenous and exogenous agents can inhibit the acquisition of genetic mutations via modulating the phase I and phase II enzymes, blocking reactive species and thus maintains the structure of DNA. So, a genetic test “AMES assay” was conducted to prove the antimutagenic activity of *M. exotica.* In the antimutagenic studies, the extract/fractions were found to be non-mutagenic that may be due to the fact that constituents does not interact directly with DNA and did not block the DNA synthesis, leading to the induction of SOS mechanism (Arif et al. 2004). Our finding of dose dependent effect of extract/fractions in Ames assay was in agreement with other findings (Aqil et al. 2008). In 2013, Zahin et al. also demonstrated the dose dependent antimutagenic activity of polyphenolic rich benzene fraction of *M. koenigii* in Ames *Salmonella* mutagenicity assay. It exhibited the antimutagenicity activity of 72–86 % against various mutagens i.e. sodium azide, methyl methanesulfonate, benzo(a)pyrene and 2-aminofluorene at maximum tested concentration for both TA98 and TA100 strains of *S.typhimurium*. In our study, ethyl acetate fraction was found to be exhibit the maximum antimutagenic potential. High amount of polyphenolic compounds in ethyl acetate fraction may contribute to its high inhibitory activity against 2AF-induced mutagenicity for TA100 strain. Modulators in the ethyl acetate fraction might interfere with the metabolic activation of 2-AF by altering the structure and function of cytochrome p-450 enzyme and providing the protection against chemically induced mutagenesis. Cytochrome p-450 enzyme catalyze the conversion of 2-AF into N-hydroxy-2-aminoflourene which interacts directly with DNA and induces the mutagenesis (Zahin et al. 2014). The present study reveals the direct inactivation of mutagen, before gene mutation occurred. In 2011, Schwarz et al. also found that estradiol-dependent cancer might be regulated by polyphenolic compounds. Mixture of antioxidants has been reported in various plants like *Castela texana* and *Stevia pilosa* with antimutagenic properties. These secondary compounds have the ability to scavenge the reactive oxygen species, reduce the mutagenicity, improve the alkylated DNA damage and therefore, halt the cellular mutability (Del-Toro-Sánchez et al. 2014). A scientific research that mainly focused on developing the phyto-originated medicine was found to be very efficient in providing prophylaxis against mutation and cancer (Sarac et al. 2015). So, the best way to reduce the increased cancer prevalence rate is the incorporation of chemopreventing agents especially of plant origin into diet (Basgedik et al. 2015).

## Conclusion

The present study shows that the *M. exotica* leaves are rich source of phenols and flavonoids. Among the different extract/fractions obtained by sequential extraction method, ethyl acetate fraction exhibited maximum antioxidant as well as antimutagenic activities. The same fraction was found to be rich in different polyphenolic and flavonoid compounds. These observations indicate the potential of the plant to be further taken up to analyze it in battery of biological assays, so that the plant can be used further in chemotherapy.
